# Unsupervised automated high throughput phenotyping of RNAi time-lapse movies

**DOI:** 10.1186/1471-2105-14-292

**Published:** 2013-10-04

**Authors:** Henrik Failmezger, Holger Fröhlich, Achim Tresch

**Affiliations:** 1 Max Planck Institute for Plant Breeding Research, Carl-von-Linné-Weg 10, 50829, Cologne, Germany; 2 Institute for Genetics, University of Cologne, Zuelpicher Str. 47a, 50674, Cologne, Germany; 3 Algorithmic Bioinformatics, Bonn-Aachen International Center for IT, Rheinische, Friedrich-Wilhelms-Universität, Bonn, Dahlmannstr. 2, 53113, Bonn, Germany; 4 Gene Center and Department of Biochemistry, Center for Integrated Protein Science CIPSM, Ludwigs-Maximilian University, Munich 81377, Germany

## Abstract

**Background:**

Gene perturbation experiments in combination with fluorescence time-lapse cell imaging are a powerful tool in reverse genetics. High content applications require tools for the automated processing of the large amounts of data. These tools include in general several image processing steps, the extraction of morphological descriptors, and the grouping of cells into phenotype classes according to their descriptors. This phenotyping can be applied in a supervised or an unsupervised manner. Unsupervised methods are suitable for the discovery of formerly unknown phenotypes, which are expected to occur in high-throughput RNAi time-lapse screens.

**Results:**

We developed an unsupervised phenotyping approach based on Hidden Markov Models (HMMs) with multivariate Gaussian emissions for the detection of knockdown-specific phenotypes in RNAi time-lapse movies. The automated detection of abnormal cell morphologies allows us to assign a phenotypic fingerprint to each gene knockdown. By applying our method to the Mitocheck database, we show that a phenotypic fingerprint is indicative of a gene’s function.

**Conclusion:**

Our fully unsupervised HMM-based phenotyping is able to automatically identify cell morphologies that are specific for a certain knockdown. Beyond the identification of genes whose knockdown affects cell morphology, phenotypic fingerprints can be used to find modules of functionally related genes.

## Background

Reverse genetics tries to unravel gene function by the examination of phenotypic effects after a gene perturbation. The rationale behind this approach is that the perturbation of genes involved in the same cellular function are likely to produce similar phenotypes. RNA interference techniques made reverse genetics an effective and cost-efficient approach. The traditional phenotypic characterization by macroscopic traits (e.g. clinical endpoints like diabetes or physiological endpoints like body weight) is complemented by traits obtained at the molecular level (e.g. gene expression-, protein-, metabolite abundances). Phenotyping of cell morphologies has been introduced as an intermediate description level which attempts to combine the advantages of both macroscopic and microscopic description levels, namely interpretability respectively high information content.

For the analysis of microscopic images, single cell images are converted into a vector of 10–200 morphological descriptors [1-4]. These morphological descriptors are sufficiently rich to distinguish various physiological states of a cell, such as mitotic and apoptotic phases [5-8]. The purpose of these methods is the clustering of cells into meaningful, phenotypically distinct classes [9,10]. Time-lapse imaging enhances the discrimination of phenotype classes by generating a dynamic view on the morphological changes, yet introduces another layer of data complexity. The amount of data generated by high-throughput microscopy requires automated analysis methods for reasons of objectivity, reliability and efficiency. Several supervised methods have been proposed in this context. Cell nuclei were classified to mitotic phases using a support vector machine [11,12] and afterwards a finite state machine [13] or an HMM is used to correct for improbable transitions between the respective phases [14].

Supervised methods depend on training data that has been labelled by an expert. They are incapable of discovering new, previously unseen phenotypes. Manual training is time consuming, depends largely on the biological knowledge and experience of the expert, and has to be repeated with each change of experimental conditions. This hampers the application of supervised methods to high throughput RNAi screens in which a large, unknown phenotypic variability is expected. It has been shown recently that unsupervised methods can accurately cluster cells in time-lapse movies to mitotic phases using an appropriate initialization to cell cycle phases and an HMM with multivariate Gaussian emission probabilities [15].

We followed this line of investigation and provide a method that automatically extracts interesting phenotypes from RNAi movies. Our method is sensitive and efficient enough to screen hundreds of movies. Apart from being able to identify known cell cycle states, we discover a representative selection of phenotypic states characterising abnormal cell morphologies. The abnormal cells of a given knockdown define a typical profile, which we use as a fingerprint for comparing different knockdowns. We find that replicate movies have similar fingerprints and that knockdowns having similar fingerprints are known to function in common pathways.

## Results and discussion

### HMM phenotyping annotates time-lapse perturbation movies

We used time-lapse movies from the public Mitocheck database [16] for high throughput phenotyping. These movies were created using siRNA microarrays. The cells on the microarray spots were transfected by siRNA and are expressing green fluorescent protein (GFP)- tagged histone 2B proteins, which mark the chromatin in the nucleus. They were incubated with the siRNA for 18 hours and afterwards tracked for 48 hours by fluorescence imaging. Every plate had 7 spots with negative controls. Every gene in the Mitocheck database was targeted by at least 2 different siRNAs on 3 spots. Mitocheck contains an enormous number of movies (about 190 000), with an average of initially 67 (±30) cells per spot [16]. A comprehensive analysis of all movies is extremely time-consuming, even for efficient methods. For the scope of this paper, we therefore pre-selected 1656 movies of 315 distinct gene knockdowns with an increased chance of exhibiting cell-cycle related phenotypic aberrations. Among these 315 genes, 44 were known to show morphological aberrations [17], 78 were cell cycle associated genes, and 63 were tumour suppressor genes, furthermore we added 130 genes that were selected at random from all genes in the Mitocheck database.

**Figure 1:**
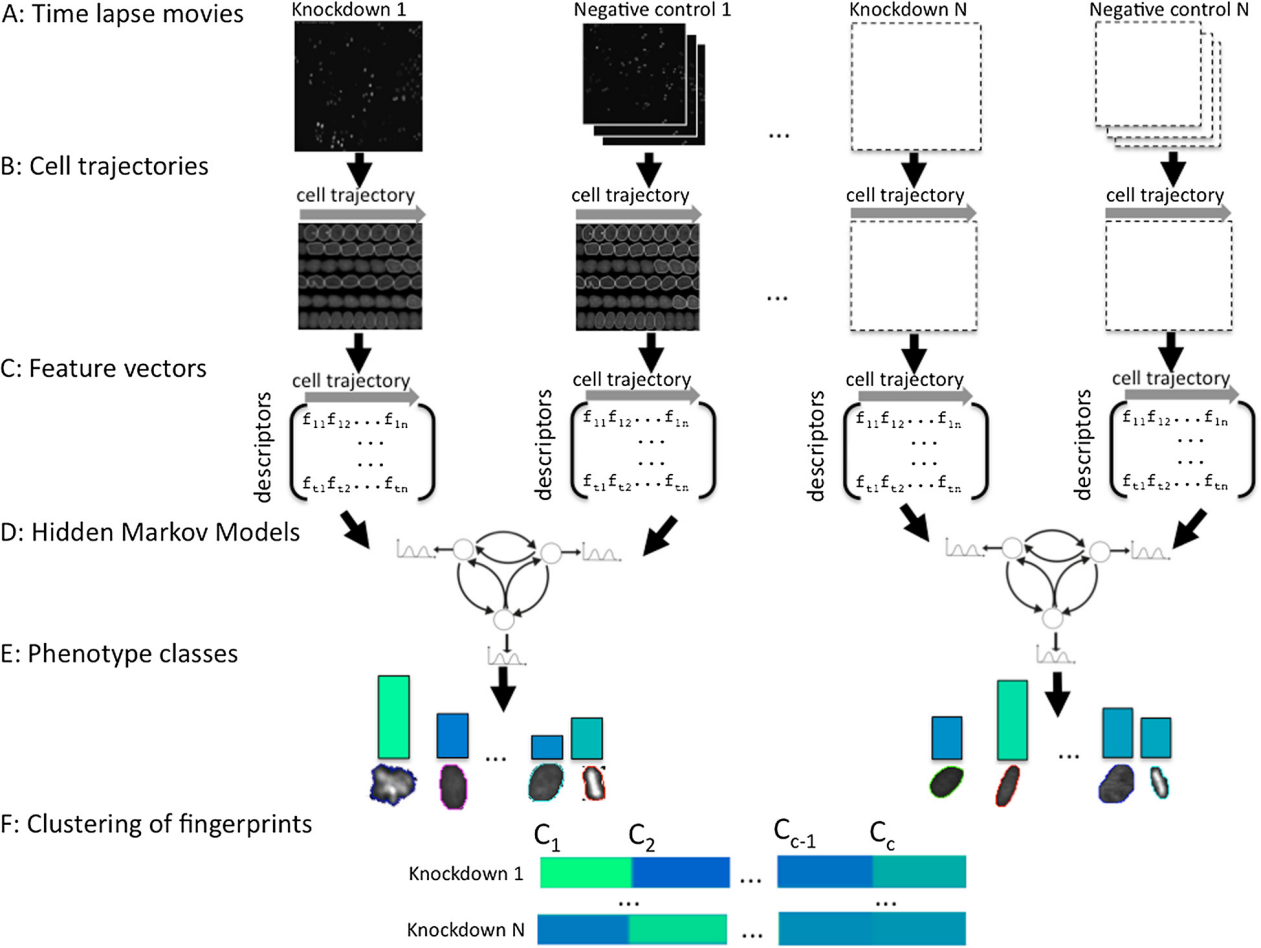
**Workflow of the method. Each knockdown movie comes along with at least three negative control movies. A**: Cells in Time Lapse Movies are segmented and identified. **B**: Tracking of the cells using CellProfiler results in trajectories of cells. **C**: Morphological features are calculated for each cell, which gives a sequence of high dimensional feature vectors for each trajectory. **D**: An HMM with Gaussian emission densities is learned on the pooled feature vectors of each knockdown – negative control pair (for clarity of presentation, the HMM has only 3 instead of 6 states actually used in the analysis). **E**: Cell morphologies are clustered to phenotype classes by the HMM Viterbi path assignment. Knockdown-specific phenotypes are extracted by comparing the phenotype class frequencies. **F**: Knockdown-specific phenotypes of many RNAi experiments are pooled and clustered by a Gaussian Mixture model. The cluster proportions of a knockdown define its phenotypic fingerprint.

The open source software CellProfiler 2.0 was used for quantitative image processing [1]. CellProfiler provides methods for the detection, segmentation and tracking of cells, and it calculates about 85 morphological features for each single cell (Figure 1A,B,C). We realized that the CellProfiler watershed algorithm for cell segmentation had a tendency to erroneously split nuclei. We therefore implemented a Cell Profiler Plugin for segmentation correction according to [18], which can be downloaded from the accompanying website http://www.treschgroup.de/movieanalysis.html. Principal Component Analysis was applied to reduce noise and to decorrelate the features (Methods). As the cells did not move substantially from one frame to another, the standard CellProfiler nearest neighbour tracker yielded good results (Methods).

The desired grouping of cells into phenotype classes can be achieved by clustering the corresponding feature vectors. Since our feature vectors are high dimensional with correlated numeric entries, a clustering based on a multivariate Gaussian mixture model would be an option. However, one would neglect the longitudinal structure of the data obtained from cell trajectories. We decided to use Hidden Markov Models with multivariate Gaussian emission distributions for phenotyping (Figure 1D, Methods). HMMs are a natural generalization of mixture models, which account for the time dependence of the observations.

An appropriate initialisation of the Gaussian distributions is important to ensure a good fit of the model to the data. Many changes in the cell phenotype are cell cycle related. Additionally, abnormal phenotypes tend to arise at certain stages of the cell cycle. Therefore, we chose a cell cycle dependent initialization. We assigned a relative cell cycle time to every cell nucleus in the trajectory (Methods). The cell cycle was then equipartitioned into 6 intervals, and the parameters of a Gaussian distribution were learned from the feature vectors in every interval by maximum likelihood estimation. Although cell cycle phases differ in their lengths, we chose equidistant intervals in order to ensure an unbiased initialisation. The learning of the HMM was done by the Baum-Welch algorithm (Methods). Each cell was then assigned a phenotype class using the Viterbi algorithm (Methods). A Principal Component Analysis (PCA) of the means of the phenotype classes helps to assess their (dis-)similarity and to tune the number of classes in the model (Additional file 1: Figure S1A), which we set to 6.

By definition abnormal phenotypes only occur in knockdowns, but not in the wild type. Knockdown movies were therefore always compared to negative controls (Figure 1). Knockdowns and negative controls were selected from identical plates to account for plate-to-plate variance. Cells from the knockdown and at least 3 adjacent negative controls were pooled for the training of the HMM (Figure 1D). This ensures that the variety of morphologies in the regular cell cycle is properly reflected by the states of the HMM. Knockdown-specific (abnormal) states (phenotypes) were identified by counting their occurrence in the trajectories of knockdown cells and in the trajectories of wild-type cells. Afterwards a fixed threshold (see Methods) was applied to decide whether a state was almost exclusively found in the knockdown and hence called abnormal (Figure 1E).

### HMM phenotyping extracts static and dynamic characteristics of a knockdown

The HMM can be seen as a data compression method, which summarizes the morphologies through discrete phenotype classes (multivariate Gaussians) and the dynamics of the cell trajectories through a transition matrix. On top of the class annotation provided by a Gaussian mixture model (GMM), the HMM performs a smoothing of the class annotations along a cell trajectory. We compared the learning performance of an HMM with that of a GMM. By visual inspection, the Viterbi path annotation of the HMM are more consistent (Additional file 1: Figure S5). Quantitatively, we compared the HMM and GMM likelihood of ten previously unseen cell trajectories after training by sequences of the same movie. The HMM consistently outperformed the GMM in all ten cases, indicating that accounting for time dependence is advantageous (Additional file 1: Figure S4, Methods).

**Figure 2:**
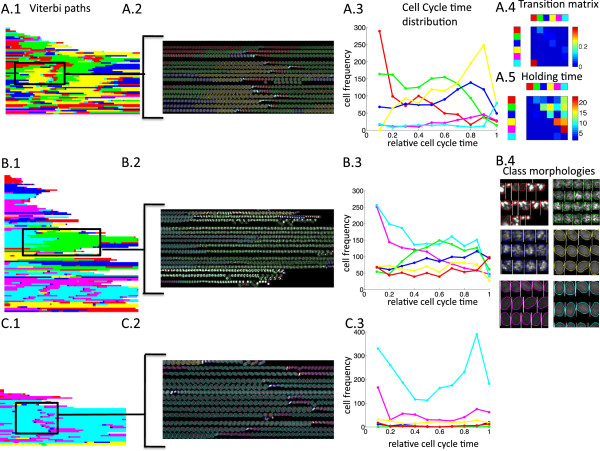
**Phenotyping of cell morphologies.** Wild type movie **(A)**, PLK1 knockdown **(B)**, PLK1 negative control **(C)**. **A.1** shows the Viterbi paths for all trajectories of a movie (paths were padded with zeros and sorted using hierarchical average linkage clustering with hamming distance, 60 trajectories). Cells labelled with their phenotype class annotation are shown in **A.2**. Cell cycle time distribution for every phenotype class is shown in **A.3**. HMM transition matrix is shown in **A.4** (diagonal entries are deleted for better visualisation). The median time a cell stays in a certain phenotype class before changing to another phenotype class is shown in **A.5**. The red state occurs early in the cell cycle whereas the turquoise state peaks at a late cell cycle time **(A.3)**. These states can be identified as the growth state after mitosis (red) and mitotic state (turquoise) **(A.2)**. The HMM transition matrix has a large probability for the transition from the turquoise to the red state. Same plots as in **A.1**–**A.3** are shown in **B.1**–**B.3** for the PLK1 knockdown (96 trajectories) and in **C.1**– **C.3** for the negative control (42 trajectories). The HMM was learned on the pooled trajectory set of both movies. PLK1 class morphologies can be seen in **B.4**. The HMM automatically extracts knockdown-specific phenotypes (**B.4**, green, blue, red state).

Based on the HMM parameters and the class annotation of each cell, we derive informative descriptors that characterize cell behaviour. A plot of the Viterbi path for every cell trajectory provides a visualisation of the phenotypic changes of a cell over time. It is, e.g., evident that the Viterbi paths of the PLK1 knockdown (Figure 2B.1) differ a lot from the Viterbi paths of the negative control (Figure 2C.1). By quantifying the relative abundances of phenotype classes in the PLK1 knockdown and in the negative control, the green, blue and red classes appear to be knockdown related, as they are virtually absent in the control (Additional file 1: Figure S3C).

Besides static phenotypes, the dynamic behaviour of the phenotype classes can be analysed. The distribution of phenotype classes along the cell cycle identifies cell cycle related phenotypes. For the wild type movie two classes are clearly associated with the cell cycle: The red class peaks at the beginning of the cell cycle and the turquoise class peaks at the end of the cell cycle (Figure 2A.3, Additional file 1: Figure S1B). From the overlay of the cell image trajectories with their phenotype class annotation, it is obvious that the turquoise and red classes represent the mitotic phase and growth phase, respectively (Figure 2A.2, Additional file 1: Figure S2). The remaining classes also show certain cell cycle time specificity, which however is less pronounced (Figure 2A.3).

The HMM transition matrix contains the transition probabilities between phenotype classes. As such, it provides information about phenotype dynamics. E.g., the transition probability from the mitotic state to the growth state is particularly large, which is in accordance with our expectations (Figure 2A.4). Notably, the first upper diagonal in the transition matrix contains, apart from the main diagonal, the largest entries. This supports the hypothesis that cells pass through a specific sequence of phenotypes during the cell cycle. The median holding time, i.e. the median time a cell stays in a certain phenotype class before changing to another specific phenotype class shows that the cells only spend a short time in the mitosis class, but remain a long time in the growth- and synthesis phase (Figure 2A.5, green, blue, yellow).

The green and blue knockdown-specific states in the PLK1 knockdown are also cell cycle related. Both their state frequencies increase with cell cycle time (Figure 2B.3, C.3). This corresponds to the fact that PLK1 has a role in mitotic spindle assembly during cell division and is therefore expected to show a mitotic effect [19]. The biological interpretation of a phenotype class is facilitated by the inspection of a representative cell sample of that class. In Figure 2B.4, cells annotated with the green and blue phenotype show a mitotic arrest, whereas cells of the red phenotype class show an apoptotic phenotype (compare [16]).

Another way of comparing the dynamic behaviour of knockdown and control is to fix the emission probabilities of the learned HMM, and to learn two new, experiment-specific transition matrices on the knockdown and control separately. There is an obvious difference in the transition matrix of the PLK1 knockdown and its wild type counterpart (Additional file 1: Figure S3A, B).

### HMM phenotyping finds and categorizes knockdown-specific phenotypes

So far, we have described a computational method to identify knockdown-specific phenotypes in a single experiment. We applied this method to movies of 315 gene knockdowns with about 6 replicates for each knockdown (Additional file 1: Table S1). Expectedly, not all knockdowns are phenotypically different from the wild type. In order to avoid false positives we only kept those knockdowns for which at least 3 replicates using at least 2 different siRNAs had a knockdown-specific phenotype. This criterion selected 67 genes that were included in the subsequent analysis (Additional file 1: Table S2, S4). This number is reasonable with regard to the results of the initial Mitocheck analysis, where 1,249 of about 21,000 genes showed a mitotic hit in the primary screen. Recall that we do not screen for pre-defined morphologies.

**Figure 3:**
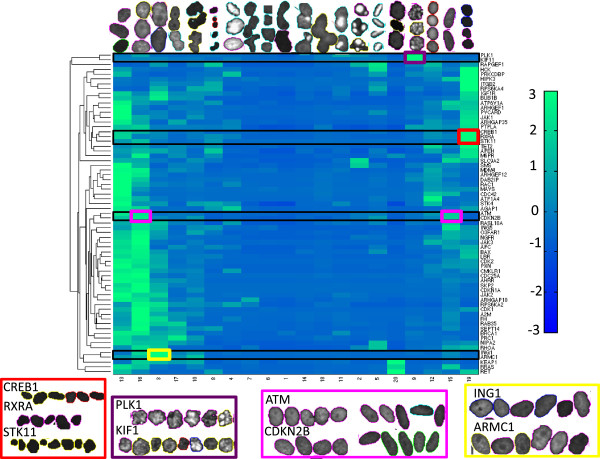
**Morphological fingerprints of those 67 genes which displayed abnormal phenotypes.** Rows indicate the affiliations of the knockdown to the 20 universal phenotype classes (upper row). Rows were arranged by hierarchical average-linkage clustering. The entries of the heatmap are z-score normalized. Knockdown-specific phenotypes show a large morphological diversity. Most fingerprints are dominated by one single cluster. Red group: CREB1 and RXRA jointly take part in the circadian clock pathway; both share a common pathway with STK11. Purple group: PLK1 and KIF11 are both associated with mitotic spindle assembly. Pink group: ATM and CDKN2B have proportions for two distinct morphologies in their phenotypic-fingerprint. They both act as cell cycle checkpoint kinase at the transition from G1 to S-phase. Yellow group: ING1 and ARMC1 have a role in metal ion transport and binding.

From each movie we only kept those cells with a knockdown-specific phenotype, which we call abnormal cells. This reduced the space of cell morphologies to phenotypes that are consequences of gene perturbations. The feature vectors of the abnormal cells were clustered using Gaussian Mixture Clustering (Figure 1F). The clusters of the Gaussian Mixture defined our universal (abnormal) phenotype classes. The phenotypic fingerprint of a gene knockdown is given by the vector of relative cluster abundances of its abnormal cells. We assume that similarity of gene function implies fingerprint similarity. Vice versa, dissimilarity of fingerprints implies distinct gene functions. A grouping of genes according to fingerprint similarity therefore cannot guarantee similar function of its group members; however, it will lead to an enrichment of functionally similar members. Note that this is an obstacle common to all feature-based approaches identifying functional similarity. We grouped fingerprints by average linkage hierarchical clustering using Euclidean distance (Figure 3, Additional file 1: Figure S6). Replicate movies of the same gene knockdown tend to cluster (Additional file 1: Figure S6), supporting the fact that morphological fingerprints are characteristic of a gene. In general, we found that fingerprints of replicates using identical siRNAs had smaller distances than fingerprints of different siRNAs targeting the same gene (Additional file 1: Table S3). It is thus beneficial to average fingerprints of replicates targeting the same gene.

The Mitocheck database categorizes cells into 16 morphological classes [16]. 20 of the 67 genes for which we found a knockdown-specific phenotype also showed at least one aberrant phenotype in the Mitocheck database (Additional file 1: Table S4). It is encouraging that some of the universal phenotype classes found by our unsupervised analysis closely resemble the morphological classes of Mitocheck. E.g. the morphologies of cells that were classified as binucleated in Mitocheck match the morphologies of cells that we assigned to cluster 17. The same holds for the Mitocheck class of large cells and our cluster 3, or for the Mitocheck class of elongated cells and cluster 15 (see Figure 3). We tested the enrichment in GO terms for our 67 genes with knockdown-specific phenotypes against the background set of the remaining 315-67=248 input genes. We found GO term enrichments for the regulation of apoptosis, protein phosphorylation, cell motility, response to stress, as well as signal transduction (Additional file 2: Table S5).

Most fingerprints are dominated by one or two universal phenotype classes (Figure 3). This suggests that certain groups of genes induce a specific phenotype. Conversely, some phenotype classes are specific for a small group of genes. These are the most interesting groups as they most likely indicate a functional relationship between their members. The genes PLK1 and KIF11 for example are clustered together and occupy a phenotype class that has large morphological differences to all other phenotype classes (Figure 3, purple cluster). PLK1 has a role in centrosome maturation and bipolar spindle formation [19]. Similarly KIF11 has a role in the formation of the bipolar spindle [20]. Genetic fingerprints thus clearly identified functionally similar genes by the extraction of knockdown-specific phenotypes and clustering.

Furthermore, the cluster composed of ARMC1 and ING1 contains large, granulated nuclei (Figure 3, yellow cluster). Both genes are associated with metal ion transport and binding (AmiGo version 1.8, [21]).

Another cluster of two joint genes (CREB1, RXRA) and one more distant gene (STK11) shows dark and very small nuclei (Figure 3, red cluster). CREB1 and RXRA are both transcription factors [22,23]. They jointly take part in the circadian clock pathway [24]. STK11 that is clustered more distant from CREB1 and RXRA is a serine threonine kinase [25]. STK11 regulates many signalling pathways in cell growth, cell polarity and cell metabolism [26] and also acts as tumour suppressor. Interestingly, STK11 negatively regulates the CREB-regulated transcriptional co-activator (CRTC) [27]. Members of the CRTC family interact with CREB1 and enhance its expression [28]. The relationship between STK11 and RXRA is more subtle, however they both have a role in the adipocytokine pathway [29,30].

An example of a knockdown-specific phenotype with two distinct morphologies is the group including the genes CDKN2B and ATM. The phenotypic fingerprints of these genes have large proportions for a round phenotype with light speckles, as well as for an elongated phenotype (Figure 3, pink cluster). Both genes are cell cycle checkpoint kinases that act at the transition from G1 to S-phase and induce G0/G1 arrest of melanoma cells [31,32].

## Conclusions

The automated extraction of morphological phenotypes in high throughput applications is a major task for future high content screens. We have developed an unsupervised method that is able to detect morphological changes in knockdown movies compared to their negative controls. This required the discrimination of phenotypes related to physiological cell behaviour from abnormal phenotypes that are consequences of perturbations like knockdowns or drug treatment. We consider our method as a dimension reduction approach for the vast space of cell images. We achieved this in two steps, first by assigning a discrete morphological state to every cell by the Viterbi algorithm, and second by grouping cells with abnormal states into universal phenotypes. Unlike supervised approaches, our method is best suited for the discovery of previously unseen phenotypes, for which prior knowledge does not exist. Nevertheless, it automatically recovers the regular phenotypes arising during the regular cell cycle.

Our method has been applied to high throughput RNAi experiments. It provides a number of informative visualizations and summary statistics, which is indispensible when dealing with big data. Most importantly, it generates a comprehensive summary of the distinct morphological states that constitute the phenotype space (Figure 3). By assigning a hidden state to each cell image, our method can even reveal the dynamic interplay between these morphological states (Figure 2).

The throughput achieved by our method allows us to perform a comparative analysis of hundreds of genes in a few days of compute time. We extract previously known as well as novel abnormal morphologies, and cluster genes according to their knockdown-specific phenotypes. RNAi induced morphological similarity of genes is a proxy of functional similarity and is thus an important step towards identifying modules of functionally related genes and the discovery of metabolic and signal transduction pathways. However, we are aware that morphological similarity alone is not a sufficient proof of pathway membership; subsequent biochemical validation is indispensable.

We mention that our method can easily be extended to multi-colour movies in which various organelles of the cell are fluorescently tagged by different dyes. In this case we expect a large increase in the diversity of pathological phenotypes. The relatively fast and cheap process of data acquisition designates this method for the large scale screening of gene-drug or gene-environment interactions.

## Methods

### Image processing

Image processing was based on the following steps: (1) cell nuclei detection and segmentation (2) morphological feature calculation and (3) tracking of the nuclei over time.

The open source software CellProfiler 2.0 [1,33] was used for cell nucleus segmentation and identification, morphological feature extraction and cell tracking. Cell nuclei were detected by Otsu thresholding [34] followed by the watershed algorithm [35] that separated clustered nuclei. As we realized that the watershed algorithm often oversegmented objects, we implemented a segmentation correction scheme according to [18]. This scheme was applied on the results of the watershed algorithm. For cell tracking, the CellProfiler 2.0 distance tracker was used. The tracking delivered good results, as the cells only moved slightly from one frame to another. Wrong associations are the most serious error when tracking cells over time. We counted wrong associations in one wildtype movie. Altogether, we only found five wrong associations in 96 trajectories.

The feature set was composed of 85 features including shape features, Zernike moments, texture features based on the co-occurrence of pixel values, and pixel intensity features. All features were calculated by CellProfiler 2.0.

All movies were acquired from the Mitocheck database. The cells in these movies were imaged for 48 hours with a time-lapse of 30 minutes [12,16].

### Data preprocessing

Principal Component Analysis was applied to reduce data dimensionality and to decorrelate features. The features were normalized by z-score standardization. The HMM was learned on the principal components that accounted for 95% of the variance in the data.

### Assignment of cell cycle time

The tracking procedure delivered the cell division time points in the trajectory. The mean duration of the cell cycle *T* was estimated as a quotient of the length of all trajectories, divided by the total number of division events in the particular movie. For cells that were observed at time *t* between two division events at times *t*_*1*_*< t*_*2*,_ we defined the relative cell cycle time *r* as quotient *r=(t-t*_*1*_*)/(t*_*2*_*-t*_*1*_*).* Cells in the trajectory before the first division event, were assigned a cell cycle time *r=1-t/*max*(t*_*1*_*,T)* with *t*_*1*_ being the time of the first division event. If cells were observed after the last division event, we defined *r=(t-t*_*n*_*)/max(t*_*w*_*-t*_*n*_*,T)* whereas *t*_*n*_ was the time of the last division event and *t*_*w*_ was the length of the trajectory. Cells that never divided during the observation period were assigned a relative cell cycle time *r =t/t*_*w*_.

### Hidden Markov Model

Hidden Markov Models are widely used for finding patterns in sequential data. In our case the HMM was applied to a sequence ***x****=(x*_*1*_*,…,x*_*t*_*)* of cell real-valued feature vectors (the input data). HMMs describe the distribution of ***x*** as generated by a set of corresponding latent state variables ***z****=(z*_*1*_*,…,z*_*t*_*)*, where each z_j_ assumes one of *N* discrete states. By assumption, the latent variables form a time-independent Markov chain.

The model is characterized by a tuple *(A=(a*_*ij*_*), to b=(b*_*j*_*), π=(π*_*i*_*))*[36]. Here, *π*_*i*_ is a vector of initial state probabilities; *a*_*ij*_ and *b*_*j*_ are probability matrices. a_ij_ includes the transition probabilities between hidden states. We assumed Gaussian emissions, so in our case b_j_=p(x_j_|z_j_)=N(x_j_;μ_j_;Σ_j_) with mean μ_j_ and covariance matrix Σ_j_.

The hidden states of the HMM define the phenotype classes of cell nuclei. The parameters of the HMM are estimated by maximum-likelihood through an Expectation-Maximization (Baum-Welch) algorithm [37].

The Viterbi algorithm calculates for every feature sequence of a cell trajectory the most likely sequence of hidden states in the Hidden Markov Model [38]. This can be seen as a dynamic clustering where the hidden states of the HMM are the cluster centres.

The number of hidden states was manually fixed to 6. We considered this number of states as on the one hand high enough to ensure that knockdown-specific phenotypes are recognized, and on the other hand as small enough to avoid joining similar phenotypes and overfitting. The cell cycle was divided into 6 time windows of identical length, and the empirical mean and covariance matrix of the feature vectors of cells in a time window were used to initialize the mean respectively the covariance matrix of a Gaussian emission distribution. The transition matrix *A* and *π* were initialized uniformly by letting *a*_*ij*_*=1/N* and *π*_*i*_*=1/N* for all *i* and *j.* The HMM is thus a fully connected graph that enables self-loops.

Numerical singularity of the covariance matrix (i.e., the determinant of the covariance matrix is close to zero) is a serious practical problem for the estimation of Gaussian distributions [39]. To avoid this we added a constant diagonal matrix with diagonal entries of 0.08 to the covariance matrix in every learning step.

In order to compare the HMM clustering with Gaussian Mixture Model clustering (GMM), we created a GMM from the HMM means and covariance matrix. We only learned the mixture proportions of the GMM. The goodness-of-fit for Gaussian emission probabilities is a monotonic function of the (log-) likelihood of the model, which has the result that HMMs will necessarily outperform GMMs in terms of goodness-of-fit. As a quality measure for HMM and GMM clustering performance we used thus the marginal probabilities of unseen cell trajectories.

### Extraction of knockdown-specific phenotypes

In order to increase variability and to avoid batch effects every knockdown movie was compared with a pooled set of three negative controls from the same plate in order to account for plate-to-plate variance. Phenotype classes (hidden states of the HMM) were assigned for every cell trajectory in all 4 movies by the Viterbi algorithm. Afterwards, knockdown-specific phenotypes were detected by comparing the proportion of a certain phenotype class in the knockdown trajectories and the trajectories of the three negative controls (Additional file 1: Figure S3C). A phenotype class had to show a proportion larger than 1.95 in the knockdown movie compared to the negative control in order to be considered as a knockdown-specific phenotype. Furthermore this phenotype class had to be present in at least 5% of the cells in the trajectories of the knockdown movie.

### Phenotypic fingerprints

Cells that were assigned a knockdown-specific state by the Viterbi algorithm were extracted.

For every knockdown at least 3 replicates had to show a knockdown-specific phenotype in order to be further processed. This check reduced the number of false positives. Cells from all knockdowns that passed the checks were pooled and Gaussian mixture clustering was applied to them. We used 20 components, which seemed to be a good choice to represent the variability in morphologies of aberrant nuclei. The phenotypic fingerprint was constituted by the relative cluster assignments of all knockdown-specific cells. Hierarchical average linkage clustering with Euclidean distances was used to group phenotypic fingerprints.

GOrilla was used for GO term enrichment analysis [40].

### siRNA scoring

For two siRNAs S1 and S2 that target the same gene we calculated the score that measured their likelihood to cluster together by:

$$\frac{\frac{1}{\left|S_{1}\right|+\left|S_{2}\right|}\left(\sum_{i,j\in S_{1}}d\left(i,j\right)+\sum_{i,j\in S_{2}}d\left(i,j\right)\right)}{\frac{1}{\left|S_{1}\right|*\left|S_{2}\right|}\sum_{i\in S_{1}}\sum_{i,j\in S_{2}}d\left(i,j\right)}$$

where *d* is the Euclidean distance between to phenotypic fingerprints.

### Performance

Image processing in CellProfiler 2.0 including cell segmentation, feature calculation and tracking takes about 1 hour 20 minutes for a movie with about 90 frames and 50 cells in the first frame on a MacBook Pro (2.2 GHz Intel Core i7, 8Gb RAM).

Our approach including HMM calculation and extraction of knockdown-specific phenotypes takes between 1.8 minutes and 5.8 minutes in dependence on how many diagnostic plots are generated.

### Implementation

CellProfiler 2.0 is implemented in Python. The Matlab implementation of Kevin Murphy (http://www.cs.ubc.ca/~murphyk/Software/HMM/hmm.html) was used for the Hidden Markov Models with Gaussian emissions. Standard Matlab functions were used for Gaussian mixture clustering and Hierarchical clustering. Matlab and Python source code is available on http://www.treschgroup.de/movieanalysis.html.

## Competing interests

The authors declare that they have no competing interests.

## Authors’ contributions

AT and HFa developed the method and performed the analysis. HF supported data analysis. AT, HFa and HF wrote the manuscript. All authors read and approved the final manuscript.

## Supplementary Material

Additional file 1 Supplemetary data. Click here for file

Additional file 2 Enriched GO terms. Click here for file
