# Supplementary material for: Unsupervised automated high throughput phenotyping of RNAi time-lapse movies
Source: BMC Bioinformatics. 2013 Oct 4;14:292. doi: 10.1186/1471-2105-14-292 (PMC3851277; doi:10.1186/1471-2105-14-292)
Supplement: Additional file 1 — Supplemetary data. [file 1471-2105-14-292-S1.pdf]

Supplementary Material

Unsupervised automated high throughput phenotyping of RNAi time-lapse movies

by Henrik Failmezger, Holger Fröhlich, Achim Tresch

Figure S1

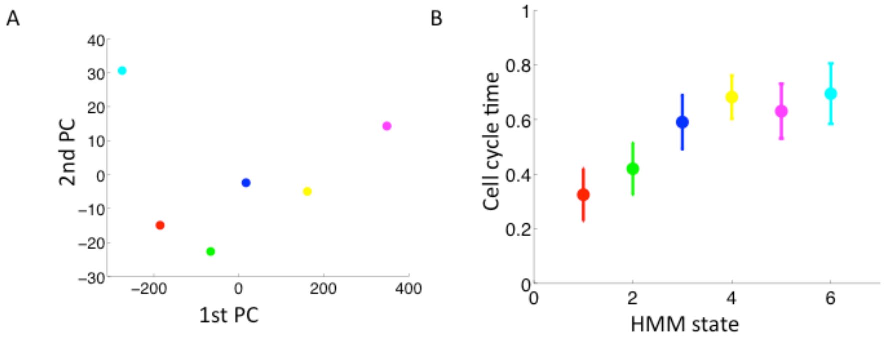

Principal Component Analysis (A) and cell cycle time distribution of phenotype classes (B). The first two principal components of the Gaussian mixture means of phenotype classes are showed in A. Mean cell cycle time and standard deviation from the mean are showed in B. The mitosis class (turquoise) is the morphologically most distant class and has the latest mean value in cell cycle time.

Figure S2

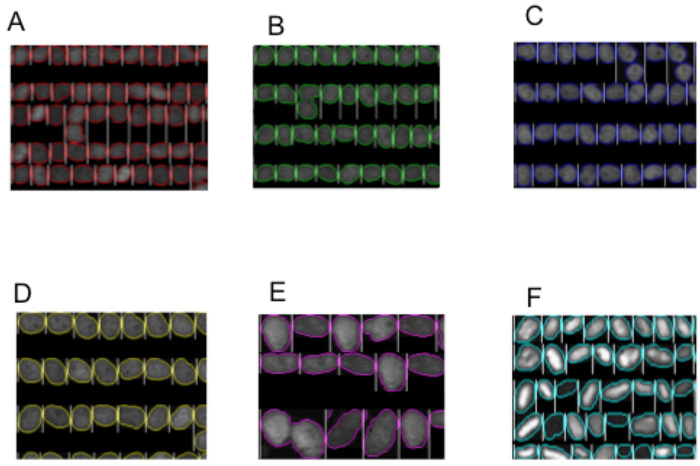

Morphologies from wild type phenotype classes that characterize different cell cycle stages.

Figure S3

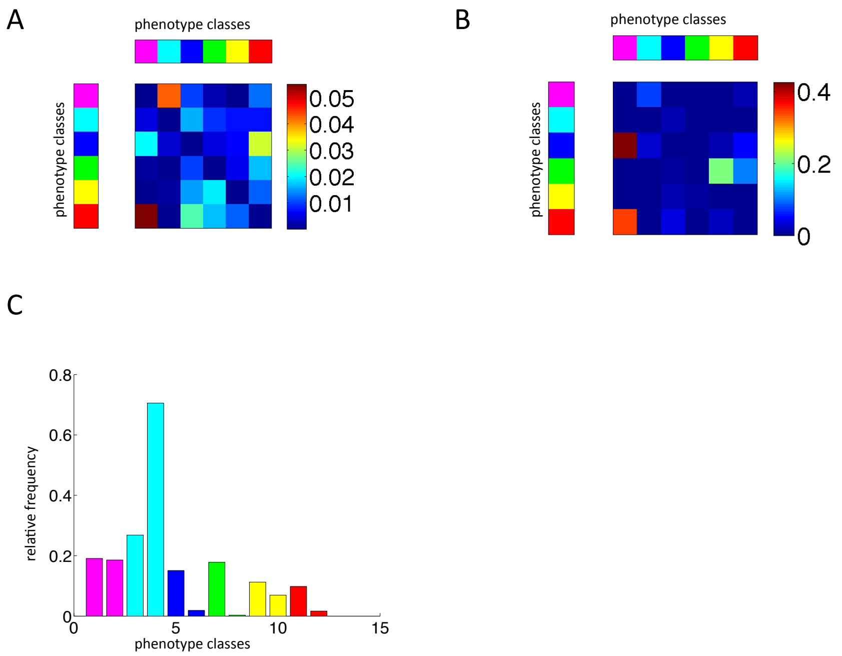

Visualisations of phenotype class transition probabilities and frequencies. Transition probability matrix of the PK1 knockdown (A) and the negative control (B). Relative phenotype class frequencies in PLK1 knockdown (left bars) and negative control (right bars).

Figure S4

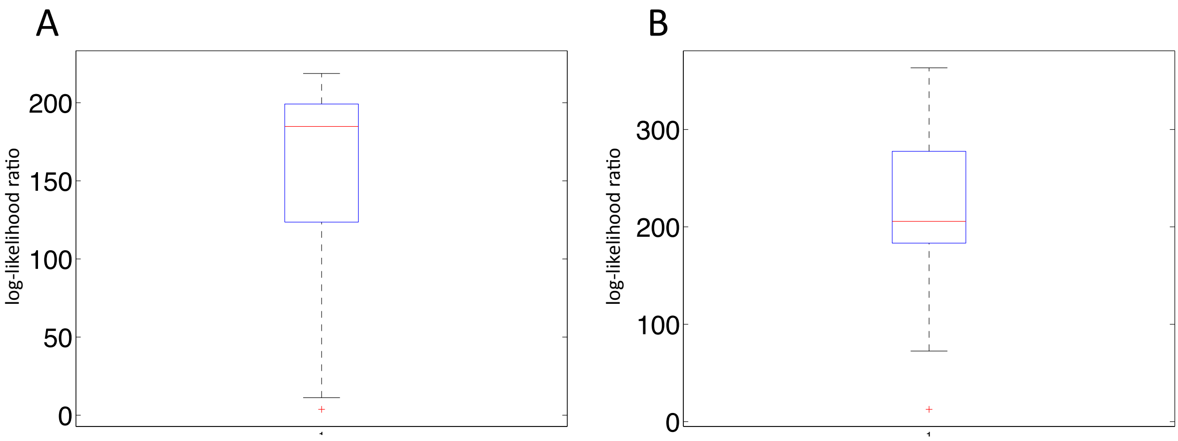

Log-likelihood rations of the HMM compared with a GMM. Covariance and means for the GMM were used from the HMM, mixture proportions were learned. Log-likelihood ratios were calculated for ten previously unseen trajectories from a PLK1 knockdown (A) and a wildtype movie (B).

Figure S5

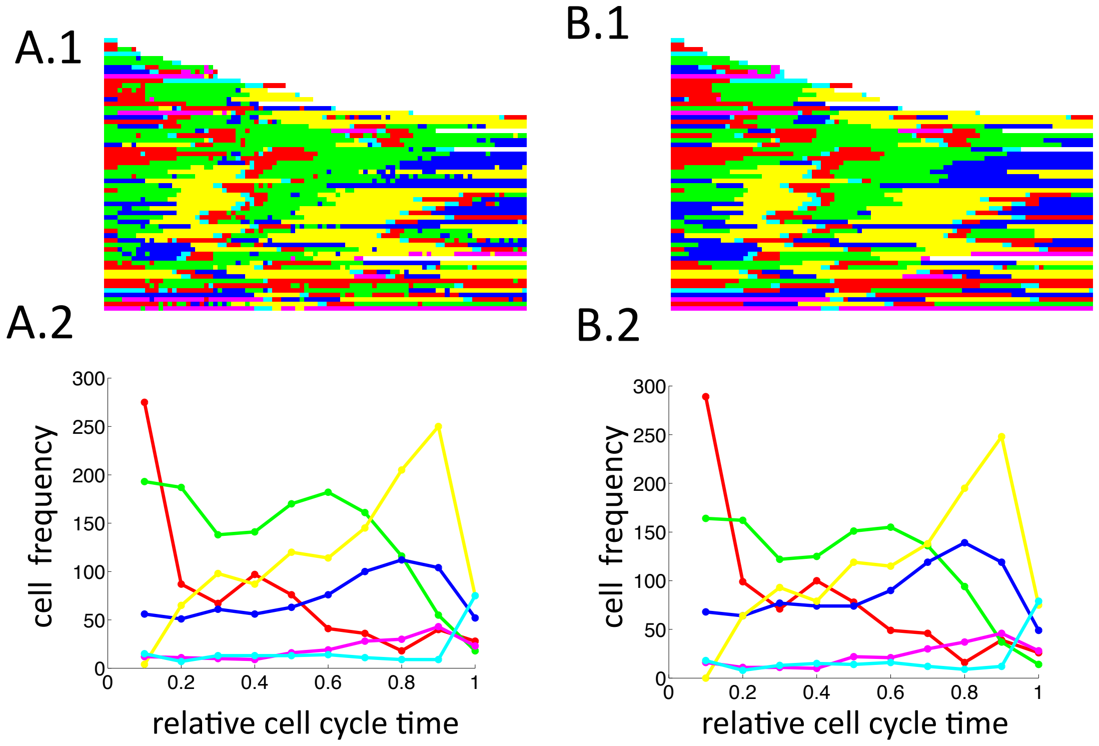

Cluster annotations by a GMM (A.1) and Viterbi paths by an HMM (B.1). Cell cycle time histograms for GMM cluster annotations (A.2) and HMM Viterbi annotations (B.2).

Table S1

A2M	MN1	CDKN2B	REN	BIN1	PLK2	FCAR	SMAD4	M6PR
ABHD2	MRPL15	CDKN2D	RET	BRCA2	PNISR	FCGBP	SMAD9	MAF
ABR	MSH2	CDS1	RGS10	BUB1B	PNN	FGD4	SMARCB1	MAFA
AC007040.10	MSN	CENPF	RGS12	C19orf56	PPIG	FGD5	SMS	MAPKAPK5
ACAP2	MTA2	CHEK2	RGS14	C1orf177	PPP2R3A	FGF6	SP1	MCM10
ACAP3	MTL5	CHN1	RHOA	C7orf25	PRC1	FH	SSH3	MDM2
ACTN4	MTSS1	CHN2	RHOB	CADM1	PRDM7	FHIT	STARD13	MDM4
AGAP2	MUTYH	CKS1B	RND1	CAMKK2	PRKCD	FKBP14	STAT3	MFHAS1
AGBL2	MYOD1	CLTC	RNF111	CAPG	PRKCDBP	FOXM1	STK11	MIS12
AHRR	NAPSA	CMKLR1	RP5	CAPNS1	PRKCI	FRG1	STK4	MKLN1
APC	NBL1	COMMD3	RPN2	CCNA1	PRR5	FRK	SUFU	MLH1
APC2	NCK1	CREB1	RPS6KA1	CCNB1	PSMC5	GHITM	SUMO3	MMP11
APEH	NDE1	CREBL2	RPS6KA2	CCND1	PTCH1	GHRHR	SYNC	
ARHGAP10	NDRG2	CREBZF	RPS6KA3	CCND2	PTEN	GSN	SYNPO2L	
ARHGAP20	NGEF	CYLD	RPS6KA4	CCND3	PTPLA	HCK	TBRG1	
ARHGAP26	NGFR	DARS	RPS6KA5	CCT7	PXN	HES1	TCHP	
ARHGAP35	NIPA2	DAXX	RRAS	CDC25A	PYCARD	HIPK3	TEAD1	
ARHGEF1	NPAS3	DCC	RTP3	CDC25B	PYHIN1	HIST1H2AB	TET2	
ARHGEF11	NPRL2	DEC1	RUNX2	CDC25C	RAB11FIP2	HMGB2	TNIP2	
ARHGEF12	NRP1	DEPDC7	RXRA	CDC37	RAB35	HOXC11	TP53	
ARHGEF16	O3FAR1	DIAPH1	SAP30	CDC42	RAP1A	HPGD	TP53INP1	
ARHGEF19	ORMDL1	DIAPH3	SASH1	CDC73	RAP1GAP	HSPB3	TP73	
ARMC1	OS9	DLEU1	SCARA3	CDK1	RAPGEF1	HTATIP2	TPBG	
ARPC1B	PAF1	DMBT1	SCEL	CDK2	RAPGEF6	IFI27L2	TRIM22	
ARPC2	PARK7	DMP1	SCN3B	CDK2AP1	RASL10A	IGF1R	TRIO	
ATM	PBRM1	DMTF1	SCUBE1	CDK4	RASSF2	IL22RA2	TSC1	
ATP1A1	PCDHB12	DNASE1L1	SDF4	CDK7	RASSF4	ING1	TSC2	
ATP1A4	PDE5A	DYNC1I1	SDHA	CDKL1	RASSF5	ING4	TSPAN8	
ATP6V0A2	PHLPP2	E2F6	SEPT14	CDKN1A	RB1CC1	IRF1	TTLL5	
ATP6V1A	PIK3C2A	EGFR	SETDB1	CDKN1B	RBMX	ITGB2	TUSC2	
ATXN3	PLEKHA5	EPHB2	SETX	CDKN1C	RBPJL	JAK1	TXNIP	
AXIN1	PLEKHF1	ERBB2	SIK1	CDKN2A	RECK	JAK2	UBTF	
BANP	PLEKHF2	ERRFI1	SIVA1	KCNG1	UHRF1	JAK3	UGT3A2	
BAX	PLEKHG2	EXT1	SKP2	KEAP1	USF2	KRT18	WSB2	
BCL10	PLEKHO1	EZH2	SLC25A11	KIAA0776	USP29	LAMC1	WT1	
BCR	PLK1	FBN3	SLC9A2	KIF11	USP33	LATS1	WWOX	
KLHDC2	VOPP1	LIMD1	ZFAND1	KIF20B	VHL	LBR	YY1	
KLHL23	VTA1	LMNB2	ZNF449	KLK10	WEE1		ZFAND1	

Gene names of processed genes.

Table S2

	All	KNSP	KNSP>=3 replicates	KNSP>=2 siRNA
#Genes	315	140	83	67
#Movies	1656	854	471	422

Number of processed genes and replicates. Genes and movies with knockdown-specific phenotype (2nd column). Genes and movies with knockdown-specific phenotypes in at least 3 replicates (3rd column), Genes and movies with at least 2 independent siRNAs below the replicates (4th column).

Table S3

Gene	Replicate Score
AGAP1	0.592374865656471
APC	0.783755299615026
ARHGAP10	1.2005
ARHGAP35	0.624253962624991
ATP6V1A	0.829597033557676
CDK1	0.598632039443441
CDK2	0.584959722095466
ITGB2	0.978303222428244
LBR	0.615701751072882
M6PR	0.558892340764823
MDM4	0.357997461468261
NGFR	0.953782131193729
NIPA2	0.318785113179696
O3FAR1	1.26931573383935
PLK1	0.362955612059338
PRC1	1.1248
PTPLA	0.457869352532224
PXN	0.334467155896423
RASL10A	0.742949956391633
TET2	0.437393195565148

Score for the affinity of siRNA replicates to fall in nearer clusters. Scores smaller zero indicate that replicates targeted by the same siRNA tend to have smaller distances. The scores were calculated for genes with at least two replicates for every siRNA only.

Figure S6

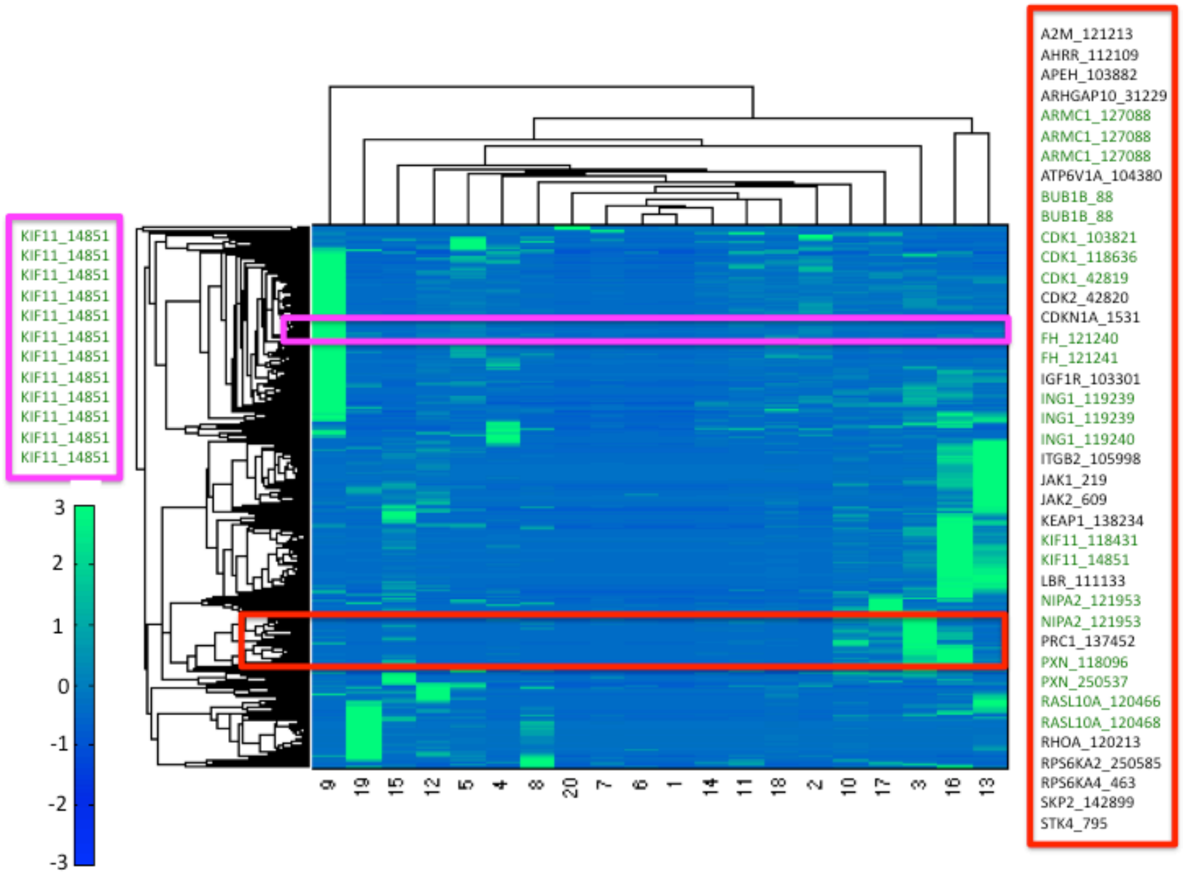

Phenotypic fingerprints of replicates. Knockdown- specific phenotypes were clustered to 20 components by a Gaussian Mixture model. The entries in the rows indicate the affiliations to the Gaussian Mixture components. Rows are grouped by hierarchical average linkage clustering, the entries in the heat map are z-score normalized. Genes that fell in a common group with at least two replicates are shown in green.

Table S4

Gen	Mitocheck phenotype	Gen	Mitochek phenotype
A2M		MAV	
AGAP1		MDM4	
AHRR		NGFR	
APC		NIPA2	
APEH		O3FAR1	
ARHGAP10		PLK1	Metaphase alignment, cell death, grape, mitotic delay
ARHGAP35		PRC1	Nuclear shape, segregation problems, binculear, mitotic delay
ARHGEF1		PRKCDBP	
ARHGEF12		PTPLA	Migration
ARMC1	Nuclear shape, segregation, polylobed, large, grape	PXN	
ARPC2	Binuclear, metaphase delay, metaphase alignment problems	PYCARD	
ATM		RAB35	
ATP1A4		RAC1	Cell death
ATP6V1A		RAPGEF1	Dynamic changes
BAX		RASL10A	
BRCA1		RET	Polylobed, dynamic changes
BUB1B	Nuclear shape, segregation, polylobed	RHOA	Migration
CDC25A		RPS6KA2	Binculear, nuclear shape, segregation problems
CDC42	Dynamic changes	RPS6KA4	
CDK1	Nuclear shape, segregation metaphase delay, binuclear	PRAS	
CDK2		RXRA	
CDKN1A		SEPT14	
CDKN2B		SKP2	
CMKLR1		SLC9A2	
CREB1		SMS	
DAB2IP	Dynamic changes	STK11	
FH		STK4	
HCK	Cell death	TET2	
HIPK3			
IGF1R			
ING1			
ING5			
ITGB2	Dynamic changes,		
JAK1	Cell death		
JAK2			
JAK3	Dynamic changes		
KEAP1	Polylobed, nuclear shape, segregation problems		
LBR			
M6PR	Nuclear shape, segregation problems, polylobed		

Genes with a knockdown-specific phenotype in our analysis and their corresponding Mitocheck phenotype.
